# Propellant Mass Gauging in a Spherical Tank under Micro-Gravity Conditions Using Capacitance Plate Arrays and Machine Learning

**DOI:** 10.3390/s23208516

**Published:** 2023-10-17

**Authors:** Shah M. Chowdhury, Matthew A. Charleston, Qussai M. Marashdeh, Fernando L. Teixeira

**Affiliations:** 1 ElectroScience Laboratory, Department of Electrical and Computer Engineering, The Ohio State University, Columbus, OH 43212, USA; 2 Tech4Imaging LLC, Columbus, OH 43235, USA; m.charleston@tech4imaging.com (M.A.C.); marashdeh@tech4imaging.com (Q.M.M.)

**Keywords:** capacitance sensors, electrical capacitance volume tomography, two-phase flow, micro-gravity mass gauging, machine learning

## Abstract

Propellant mass gauging under micro-gravity conditions is a challenging task due to the unpredictable position and shape of the fuel body inside the tank. Micro-gravity conditions are common for orbiting satellites and rockets that operate on limited fuel supplies. Capacitance sensors have been investigated for this task in recent years; however, the effect of various positions and shapes of the fuel body is not analyzed in detail. In this paper, we investigate this with various fill types, such as annular, core-annular, and stratified fills at different positions. We compare the performance among several curve-fitting-based approaches and a machine-learning-based approach, the latter of which offers superior performance in estimating the fuel content.

## 1. Introduction

Accurate in-space microgravity propellant mass gauging is critical for interplanetary spacecraft mission planning and propellant leak detection. Conventional propellant measurement techniques, however, are typically only applied when the fluid is in a settled state during thrusting maneuvers, can accumulate error over time, and are unable to detect leaks [1]. Microgravity propellant mass gauging is an active area of research at NASA in order to monitor real-time propellant mass throughout the life of the mission [2]. Due to the various possible unsettled and surface-tension-dominated microgravity fluid configurations, this remains an unsolved problem despite years of research into various techniques. For example, ideal gas-law-based techniques such as PVT gauging are susceptible to pressure sensor inaccuracy/drift and decrease in accuracy as propellant mass diminishes [3]. Thermal propellant gauging has increased accuracy at low mass levels but has a very slow response rate and makes assumptions on fluid position that may not apply in all cases. Acoustic or radio frequency modal gauging methods are currently under development but require detailed understanding of the fluid configuration and ground-based simulations [4,5]. A technique that can accurately measure real-time propellant mass and distribution without prior knowledge of fluid orientation is necessary for the success of future interplanetary missions [6].

Electrical capacitance volume tomography (ECVT) has recently received significant interest as a microgravity mass gauging technique [7,8,9]. An ECVT system is comprised of lightweight metal plates wrapped around the region of interest (RoI) and a high-speed acquisition device featuring real-time operations, ideally suited for in-space mass gauging. ECVT is also non-intrusive and non-hazardous, another set of highly desirable features for space applications. ECVT originates as a measurement tool for industrial multiphase flows, such as gas–solid, air–water, etc. [10]. The mutual capacitance data among the metal plates contain information regarding the mass distribution in the RoI, which can be used to determine the total mass fraction as well as perform imaging and velocimetry of the flow [11,12]. ECVT measurements are relatively less sensitive to the position and the shape of the material mass in the RoI, which can be further corrected via data processing.

In this paper, the difficulties in mass fraction estimation at micro-gravity conditions, originating due to the unpredictable shape and position of the fuel mass, are addressed with an ECVT sensor. Different fuel shapes and positions are considered, such as annular, core-annular, stratified, etc., commonly occurring at micro-gravity conditions. The estimation is performed through a number of techniques, such as averaging, weighted averaging, and 2-norm of the capacitance data, as well as imaging and machine learning. Also, each technique is discussed for its complexity and accuracy. It should be noted that machine-learning-based techniques have found numerous applications in flow parameter estimation [13], flow regime identification [14], and imaging [15,16,17] in electrical capacitance tomography, which is attributed to the ability to handle complex nonlinearities with machine learning algorithms given that enough training data are available.

The remainder of the paper is organized as follows. In Section 2, further discussion is provided about the ECVT sensor used here, followed by the different shapes and positions of the fuel mass, hereafter referred to as the ‘fill type’. In Section 3, a number of mass fraction estimation methods are discussed along with their respective performances. In Section 4, experimental results are provided for the machine-learning-based method. In Section 5, the main findings are summarized.

## 2. Sensor Description and Fill Types

**Figure 1:**
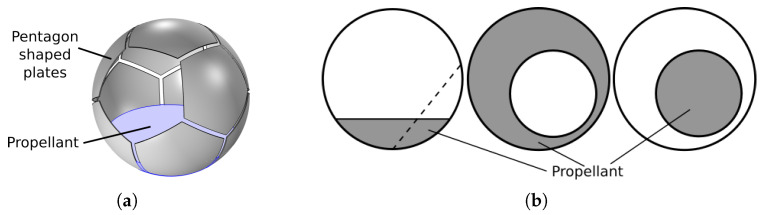
(**a**) A 12-plate spherical capacitance sensor model with stratified filling. Sensor diameter is 9.5 inches. (**b**) Different types of fills: stratified, annular, and core-annular. The stratified fill may exhibit different orientations, as indicated by the dotted line. Two distinct orientations are centered at a ‘plate’ or centered at the ‘gap’ between three adjacent plates.

An ECVT sensor consists of a tessellation of metal electrode plates around the surface of a region of interest (RoI). Figure 1a shows the sensor used here, which is of spherical shape with pentagonal plates, similar to a soccer ball. This mimics the spherical fuel tanks usually used in spacecrafts. The sensor consists of 12 equal-sized plates, a geometry called a spherical dodecahedron. The intersection region among three adjacent plates is referred to as a ‘gap’ here. There are 20 gaps for the present case. The plates are connected to a data acquisition device, which excites the plates in a sequential order and records the mutual capacitance among all possible plate pairs. The total number of measurements for an *n*-plate sensor is therefore provided as M=n2=n(n−1)2, which is 66 for the present 12-plate sensor. The mutual capacitance between two plates i−j is defined as

(1) $$C\left(i,j\right)={\left.\frac{\left|i_{i}\right|}{\omega \left|v_{j}\right|}\right|}_{v_{k}=0\;\;\mathrm{for}\;\;k\neq j}$$

where $i_{i}$ denotes the measured current at plate *i*, $v_{j}$ denotes the applied voltage at plate *j*, and ω denotes the angular frequency of the excitation signal. The rest of the plates are grounded during this process. A plate pair is often referred to as a ‘channel’. Each channel is normalized with respect to empty and full capacitance data as

(2) $$C_{\mathrm{Norm}}=\frac{C-C_{\mathrm{Empty}}}{C_{\mathrm{Full}}-C_{\mathrm{Empty}}}.$$

Here, $C_{\mathrm{Empty}}$ and $C_{\mathrm{Full}}$ refer to the capacitance data when the sensor is filled with the low- and high-permittivity materials, respectively, which are the air, $\varepsilon_{r}=1.0$, and the propellant, $\varepsilon_{r}=2.2$, for the present case. A partially filled fuel tank acts as a two-phase flow medium. The two-phase capacitance data, *C*, contain information regarding the mass distribution in the RoI, from which the total mass fraction can be estimated through an appropriate inversion algorithm. It should be noted that two-plate sensors have been deployed for mass fraction estimation [18]; however, they are suitable for rotationally stable flow regimes only. In micro-gravity conditions, the fluid configuration changes significantly, as illustrated in Figure 1b, resulting in signal changes as a result of fluid position changes at constant fluid mass. An algorithm relying on only two plates or one data channel would inevitably misinterpret this as changes in mass fraction, producing erroneous results. This would become worse if the fuel mass is close to the gap between the plates, the region with the highest sensitivity. With additional plates, additional information is available for an algorithm to determine if changes in signal are due to changes in fluid mass or fluid position, or both, producing more accurate estimations. Also, a higher number of plates offer imaging capabilities in addition to mass fraction estimation, which provides complete information of the RoI. Moreover, it is possible to synthesize a two-plate sensor out of *n* plates by electronically connecting the physical plates [19]. The dodecahedron geometry shown in Figure 1a is less sensitive to fluid position changes as compared to an octahedron, as reported in [20]. This particular design has a high degree of rotational symmetry and a good balance between electrode surface area and number of electrodes, which helps in maximizing signal-to-noise ratio (SNR) and minimizing the positional sensitivity of capacitance data, thus improving estimation accuracy. This optimization performed at the sensor design stage has reduced many complications at the data inversion step that may have occurred otherwise.

Different fill types may occur under micro-gravity conditions, such as stratified, annular, and core-annular, as shown in Figure 1b. The ‘stratified’ fill, also known as ‘settled’, may occur under thrusting conditions. The fuel mass may settle at different directions, as indicated by a dotted line in the figure. Two possible positions are considered here for this fill type: settled on a ‘plate’ and settled on a ‘gap’. The annular fill may occur when the fuel sticks to the inner wall of the tank, with possible off-centered conditions, as shown in the figure. Lastly, the core-annular fill may occur when the fuel mass floats inside the tank, with possible off-centered conditions. It is difficult to obtain micro-gravity conditions on Earth. Two common methods for that purpose include drop-tower tests and parabolic airplane flights [7,21]. However, computational techniques allow simulating such conditions with great efficiency and accuracy. To this end, the COMSOL Multiphysics 6.0 software is used here to simulate the various fill cases at different mass fractions. A complete list of the different fill types is discussed later in Section 3.5.

## 3. Different Approaches for Mass Fraction Estimation

Mass fraction estimation is a two-step process: prepare a mass fraction to a capacitance data map for different fill types, then use the map to estimate the former given the latter. Although simple, the implication of this approach is twofold. First, it requires the determination of the appropriate fill type from the capacitance data, and then a back projection may be applied to estimate the mass fraction. This is a complex problem because of the necessity to consider a very large number of fill types. This leads to a machine learning (ML)-based approach in which an ML model is trained on known fill types and used later for estimation purposes with unknown data. However, generating the large dataset for training the model requires time. On the other hand, it is possible to develop quicker methods based on the most ‘extreme’ fill types possible, acting as the basis for all others. Four such fills are the stratified case with plate- and gap-centered settlements and the fully centered annular and core-annular cases. In this section, a few quick estimation approaches based on averaging these four extreme cases are considered first along with prediction performances. Later, an ML-based approach is presented considering a total 316 fill types, including the 4 extreme ones.

**Figure 2:**
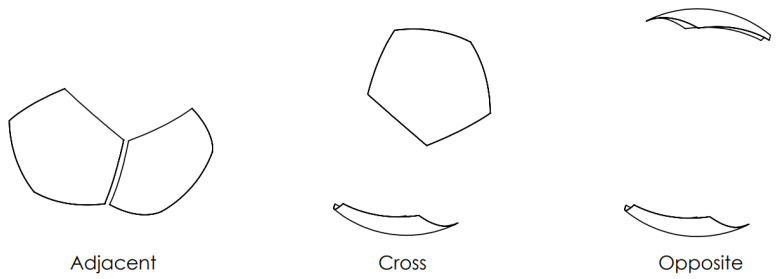
Channel types based on rotational symmetry of a 12-plate spherical ECVT sensor.

Before proceeding, the sensor responses to the four extreme fill types are discussed. Because of symmetry, the current sensor features three characteristic channel types, which are the adjacent, the cross, and the opposite, as shown in Figure 2. The adjacent channels are the ones having plates next to each other, whereas the opposite channels have a plate at the antipode of the other. The rest of the channels are categorized as cross channels.

**Figure 3:**
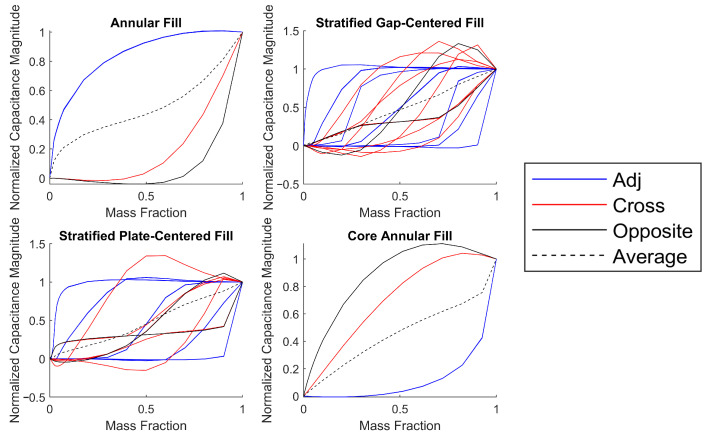
Capacitance response to different fill types, sorted by channel types.

The responses for the three channel types to mass fraction variations are shown in Figure 3 for the four extreme fill types. Being completely rotationally symmetric, the responses from the annular and core-annular fills all sort into three distinct lines according to channel types. The stratified responses present a more chaotic plot, with fewer symmetrical relationships. Additionally, the stratified cases have signal levels that peak above the normalized values as the fluid moves across regions with negative sensitivity. Despite the more irregular responses, the stratified cases average out to be very linear, while the annular and core-annular cases do not. This presents a difficult challenge in mass fraction reconstruction and fluid location classification. Next, four quick mass fraction estimation methods are presented based on these extreme fill types, followed by a comprehensive machine-learning-based approach. The mass fraction is denoted as α, ranging in [0,1], whereas the estimated mass fraction is denoted as $\hat{\alpha }$. The set of capacitance data for all the plate pairs is denoted as C.

### 3.1. Average-Capacitance-Based Approach

The simplest method of evaluating the mass fraction is to take an arithmetic average of all the channels, as illustrated in Algorithm 1. This method has the highest error due to fluid position as fluid position is simply ignored. Although not ideal for mass gauging, the averaged response of a sensor is an important metric for evaluating the stability of a given sensor design to fluid position.
**Algorithm 1** Steps for average-capacitance-based mass fraction estimation.1:**procedure** Average-Capacitance-Based Approach(α,C)2:    Normalize each channel *n* using (2), providing $C_{\mathrm{Norm}}\left(n\right)$.3:    Average the normalized channels together as $C_{\mathrm{Avg}}=\left(\sum_{n=1}^{N}C_{\mathrm{Norm}}\left(n\right)\right)/N$, where *N* is the total number of channels.4:    Build a mass fraction α to average capacitance $C_{\mathrm{Avg}}$ map for each fill type in the range [0,1].5:    Fit a polynomial to the different maps.6:    Determine mass fraction $\hat{\alpha }$ for an unknown observation using the fitted polynomial.7:**end procedure**

**Figure 4:**
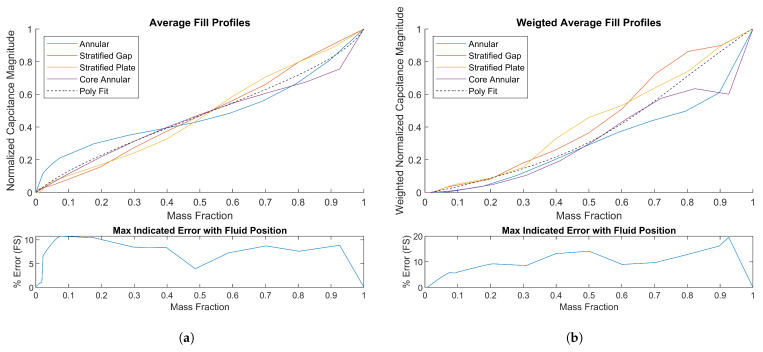
(**a**) Average capacitance response to different fill types. (**b**) Weighted average capacitance response to different fill types.

The average responses of all channels to four of the most extreme fill types are plotted in Figure 4a. The relevant error metric is the range of possible real mass fractions for a given indicated mass fraction. To calculate this, a best fit polynomial is applied to all four curves combined. This polynomial represents the theoretical calibration curve. For a given average measured capacitance, a table lookup with this curve will indicate a mass fraction. The maximum possible difference between the indicated and the real mass fraction at a given capacitance signal level is plotted as the error, as a percentage of the full scale of 1. Because different channel types have different characteristic sensitivity, this approach can potentially be improved by considering only a subset of channels in the average, e.g., where *n* is only cross channels. Also, it can be seen from Figure 4a that the stratified fill cases have a very linear response. The majority of the error in the mass fraction calculation is due to the overpowering influence of adjacent plates for low-volume annular fills and high-volume core-annular fills. When considering all fluid positions, the error band is around 10% across all mass fractions. Weighting the adjacent channels lower than the others could potentially improve the results and knowledge of the fluid position via another input could further improve accuracy.

### 3.2. Weighted Average Capacitance by Channel Type Approach

The three characteristic channels have distinct responses to fluid fills, as shown in Figure 3. As such, they can be sorted into categories based on channel type and weighted to improve the overall linearity of the response and reduce the effect of fluid position. The relative impact of a mass of fluid on the average capacitance changes as the fill level changes, for example, with adjacent channels over-contributing to the average signal at low fill levels. Therefore, the proposed weighting algorithm varies the weight of a channel type based on fill level.

A weighting algorithm is applied using the method of Algorithm 2. The *i*, *j*, and *k* parameters are determined as 6.3, 3.9, and 2.1 through iteration, which provides a combination with low error. The resulting fill profiles are plotted in Figure 4b.
**Algorithm 2** Stepsfor weighted average-capacitance-based mass fraction estimation.1:**procedure** Weighted Average-Capacitance-Based Approach(α,C)2:    Normalize each channel *n* using (2), providing $C_{\mathrm{Norm}}\left(n\right)$.3:    Sort channels into categories by type $n_{\mathrm{adj}},n_{\mathrm{cross}},and\;n_{\mathrm{opposite}}$4:    Average weighted normalized channels together, where *N* is the total number of channels
$C_{\mathrm{WeightedAvg}}=\left(\sum_{n=1}^{N_{\mathrm{adj}}}C_{\mathrm{Norm}}{\left(n_{\mathrm{adj}}\right)}^{i}+\sum_{n=1}^{N_{\mathrm{cross}}}C_{\mathrm{Norm}}{\left(n_{\mathrm{cross}}\right)}^{j}+\sum_{n=1}^{N_{\mathrm{opposite}}}C_{\mathrm{Norm}}{\left(n_{\mathrm{opposite}}\right)}^{k}\right)/N$5:    Re-normalize with respect to the full capacitance, also passed through the preceding step.
$$C_{\mathrm{WeightedAvgNorm}}=C_{\mathrm{WeightedAvg}}/C_{\mathrm{WeightedAvg},\;\mathrm{Full}}$$6:    Build a mass fraction α to the weighted average capacitance $C_{\mathrm{WeightedAvgNorm}}$ map for each fill type in the range [0,1].7:    Fit a polynomial to the different maps.8:    Determine mass fraction $\hat{\alpha }$ for an unknown observation using the fitted polynomial.9:**end procedure**

Using this weighting method, the curve shapes are more similar at low mass fractions at the cost of increased error at higher mass fractions, maintaining less than 6% error at mass fractions under 10%. A smooth transition from Weighted Averaging to the Averaging method at around 30% fill could take advantage of the low errors regions of both methods. However, to decrease the error further, it is apparent that more sophisticated approaches that rely on fluid location classification are required.

### 3.3. Linear Back Projection Image Voxel Mean Approach

The mass fraction can be derived as a byproduct of the imaging stage. That said, this method generates a 3D image of the permittivity distribution using the simplest image reconstruction method of linear back projection (LBP). Given a sensitivity matrix S and capacitance vector C, LBP is implemented as $g=S^{T}C$, where g is the image representing the mass distribution in the RoI. Then, mass fraction is calculated by averaging g over the RoI as illustrated in Algorithm 3. Due to variations in sensitivity throughout the RoI, the measured response of a plate pair can vary based on fluid position. Since ECVT image reconstruction such as LBP is an ill-posed problem, additional errors are incurred when mass fraction is calculated from the image. Therefore, this procedure is usually more prone to errors than other approaches.
**Algorithm 3** Stepsfor linear back projection voxel mean (LBP-VM)-based mass fraction estimation.1:**procedure** LBP-VM-Based Approach(C,S, D)2:    Normalize each channel *n* using (2), providing $C_{\mathrm{Norm}}\left(n\right)$.3:    Reconstruct image using Linear Back Projection as $g=S^{T}C$.4:    Apply mask D to image as $\hat{g}=gD$ so only voxels inside the RoI are included.5:    Calculate mean of the image over the voxels, providing the mass fraction as $\hat{\alpha }=\left(\sum_{n=1}^{N}{\hat{g}}_{n}\right)/N$.6:**end procedure**

**Figure 5:**
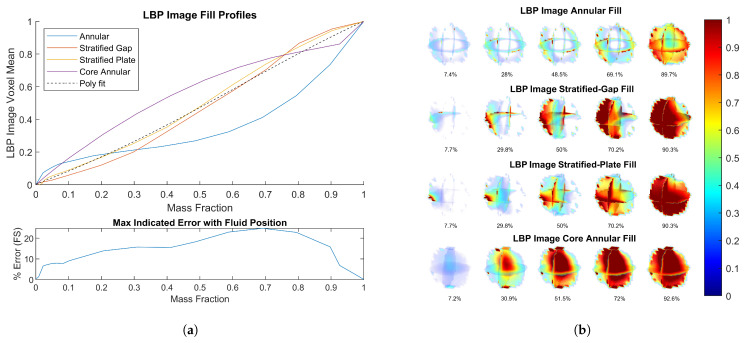
(**a**) LBP-VM response to different fill types. (**b**) LBP image reconstruction of different fill types.

The response to various fill types using LBP-VM reconstruction is shown in Figure 5a. The stratified cases are highly linear, but the sensitivity matrix struggles to account for the extreme annular and core-annular fill cases, leading to a relatively high error in the indicated mass fraction. Despite the poor mass fraction accuracy, the images generated using LBP in Figure 5b clearly show the fluid distribution. Knowledge of the fluid distribution via LBP image reconstruction could be used in combination with another method in an iterative process to improve the mass fraction estimation.

### 3.4. Capacitance-Norm-Based Approach

**Figure 6:**
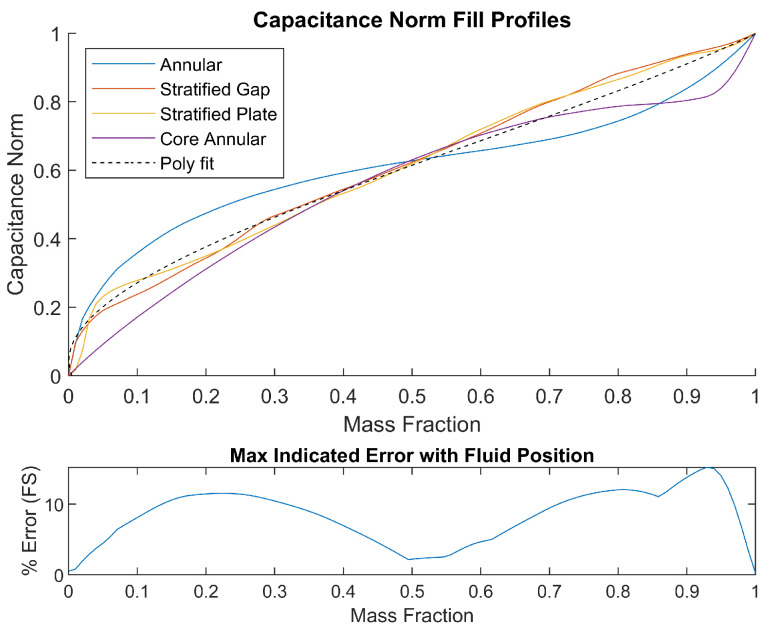
2-norm of capacitance data for various fill types and error in mass fraction estimation based on the average capacitance norm curve.

The 2-norm of the normalized capacitance vector, expressed as ∥C∥, corresponds to the Euclidean length of the capacitance vector. It is a good indicator of the mass fraction in the RoI. The norm plots for the different fill cases are presented in Figure 6. A higher norm value corresponds to higher mass fraction and vice versa. Here, the expression ∥C∥ is normalized with respect to the 2-norm of an all-ones vector of equal length so that it is bounded in [0,1]. This helps in generalizing the mappings for estimation of mass fractions. The procedure for estimation of mass fraction based on norm of capacitance data is illustrated in Algorithm 4.
**Algorithm 4** Steps for capacitance-norm-based mass fraction estimation.1:**procedure** 2-norm-of-Capacitance-Based Approach(α,C)2:    Normalize each channel *n* using (2), providing C.3:    Determine 2-norm of the capacitance vector ∥C∥ for different fill types and mass fraction values α.4:    Build a mass fraction to capacitance norm map for each fill type in the range [0,1].5:    Normalize each data point as ∥C∥/∥1∥, where 1 is an all-ones vector equal in length to C.6:    Fit a polynomial to the different maps.7:    Determine mass fraction $\hat{\alpha }$ for an unknown observation using the fitted polynomial.8:**end procedure**

The mass fraction to capacitance norm maps for different fill cases are shown in Figure 6. It is observed that the curves are non-overlapping to a significant degree, except near the mid range. This creates a problem in mass fraction estimation based on a single fitted polynomial. The maximum estimation error for the different fill types is shown below the maps in the same figure, which is primarily significant at the low mass fraction region. The norm-based procedure is simple and fast; however, it is prone to error when the fill type changes.

### 3.5. Machine-Learning-Based Approach

In this section, a *Random-Forest*-based machine learning (ML) method from the Scikit-Learn [22] Python library is used for the estimation of mass fraction. The input to this method is the normalized capacitance data as provided in (2). The methods described in previous sections are mostly limited to a few fill types due to the manual curve fitting approach. With machine learning, it is possible to incorporate many different fill types with little to no effort in parameter tuning. Moreover, if the dataset is sufficiently large, a very good performance is achieved. The first requirement of an ML approach is a sufficiently large dataset including all possible fill types. The fill types shown in Figure 3 are chosen as the dataset here, as listed in Table 1. The first two columns contain positional information of an object in the RoI in spherical coordinates (r,θ,ϕ). For the symmetric fills such as the stratified, annular, and core-annular, the radial distance *r* is irrelevant. For the stratified fill, the (θ,ϕ) indicate the orientation of the normal vector to the stratified plane. There are 26 such directions, which point towards 6 of the plates and the 20 gaps of the dodecahedron sensor. Each plate has an opposite plate, so only 6 directions are sufficient for the 12 plates. The annular and the core-annular fills are fully symmetric so there is only 1 possible orientation. The mass fractions for these three fills range in [0,1], which is divided into 100 data points, providing a total number of data points of 2600 for the stratified and 100 for the two others each. The positions for the off-centered annular and core-annular fills are described by all three coordinates (r,θ,ϕ). Here, *r* is radial distance from the sensor origin given as a fraction of the sensor radius. The (θ,ϕ) indicate the direction of the vector passing along the object center. Further, 32 possible directions are considered for each *r*, which are along the 12 plates and the 20 gaps. The mass fraction range for these fills varies depending on *r*, as observed from the table. The number of data points for each range is chosen such that roughly 1 data point per 0.01 mass fraction is maintained. The last row of the table contains the column totals. There are a total of 316 fill types considered for this section as opposed to only 4 for the previous sections. The total number of data points is 11,280, a reasonable number for ML purposes.

Next, the dataset is divided into five equal sets, of which four are used as the training sets and one is used as the test set. It is important to include data over the entire range [0,1] in each set. A possible distribution would look like {0,5,…95}, {1,6,…96}, {2,7,…97}, {3,8,…98}, and {4,9,…99}. The ML model is trained over the training set and a 4-fold cross-validation is performed to evaluate model performance. Lastly, the model is tested with the test set to evaluate the final performance. The methodology is illustrated in Algorithm 5 below.
**Algorithm 5** Steps for machine-learning-based mass fraction estimation.1:**procedure** Machine-Learning-Based Approach(α,C)2:    Generate capacitance data C by simulating different fill types over the mass fraction range [0,1].3:    Normalize capacitance data with (2).4:    Divide the data equally into five sets, of which use four for training and the remaining one for testing.5:    Train the ML model with the training set.6:    Perform a 4-fold cross-validation over the training set to evaluate model performance.7:    If satisfactory performance is achieved, test final performance with the test set.8:    If test set performance is satisfactory, the modeling is completed.9:    Deploy the model for the estimation of mass fractions $\hat{\alpha }$ for unknown observations.10:**end procedure**

**Figure 7:**
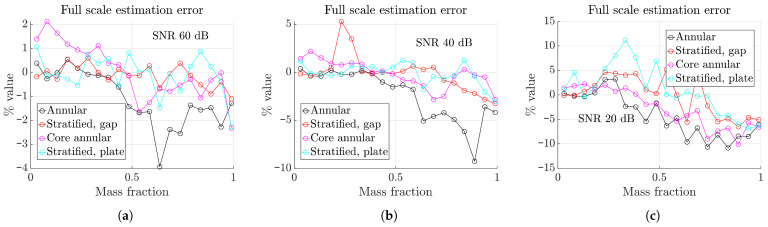
Estimation error for different noise levels as percentage of full scale of 1. (**a**) Mild as 60 dB. (**b**) Moderate as 40 dB. (**c**) Heavy as 20 dB.

At the final evaluation step, the test set is subjected to three different noise levels: mild as 60 dB, moderate as 40 dB, and heavy as 20 dB. The noise level is defined here as

(3) $${\mathrm{SNR}}_{\mathrm{dB}}=20\log_{10}\frac{\mu }{\sigma }$$

where μ and σ denote the mean and the standard deviation of the data. Standard deviations of 0.1, 1, and 10 % of the mean correspond to 60, 40, and 20 dB of noise levels, respectively. The full scale estimation errors, defined as the difference in the estimated to the actual mass fractions, are shown in Figure 7 for the four standard fill types. It is observed that the maximum error value is within 4 % for the mild case, which is comparable to the other methods described above and shows a significant improvement. For the other two noise levels, the maximum level stays within 10 and 15 %. It should be noted that 60, 40, and 20 dB are considered as good, typical, and extreme cases for capacitance measurement and tomography applications.

**Figure 8:**
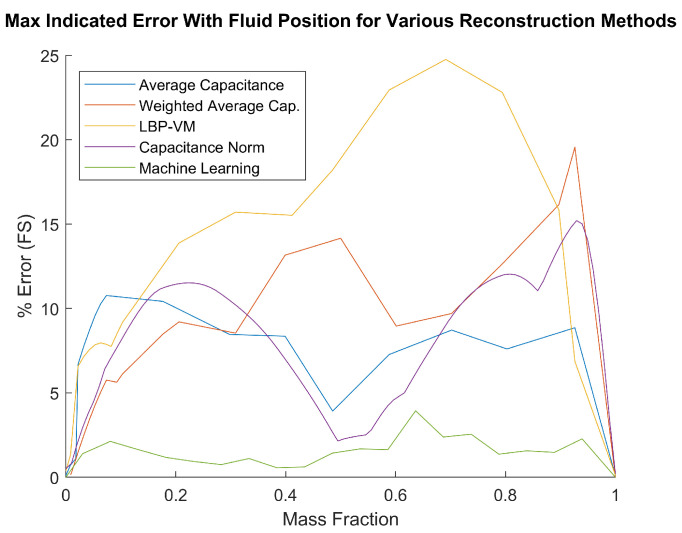
Estimation error comparison among different methods. The machine-learning-based approach exhibits the lowest error overall.

Finally, a comparison among the different methods is shown in Figure 8. Each curve there indicates the maximum estimation error among the different fill types at a given mass fraction. It is observed that the machine-learning-based method exhibits the lowest error overall, in agreement with what is concluded above. It is noted that the 60 dB noise case is chosen for the machine learning method in this graph.

## 4. Experimental Results

**Figure 9:**
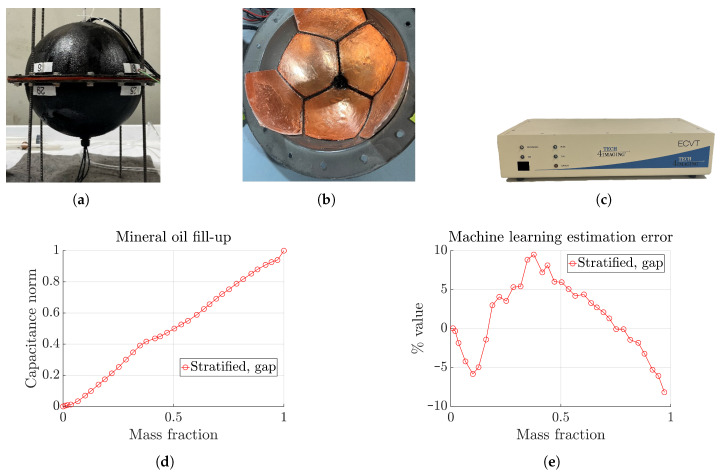
Experimental results. (**a**) Constructed spherical tank. (**b**) 3D-printed plates epoxied internal to the tank. (**c**) Tech4Imaging multi-channel data acquisition system (DAS). (**d**) Capacitance norm as mass fraction changes for a stratified filling experiment with mineral oil. (**e**) Machine learning estimation error as percentage of full scale of 1.

The spherical dodecahedron tank has been constructed for testing the real-world performance of the proposed machine learning approach. However, at present, it has only been tested with a stratified filling experiment. More realistic experiments at micro-gravity conditions are underway. The tank is constructed by bolting two flanged acrylic hemispheres, as shown in Figure 9a. The plates are constructed of formed copper sheet metal epoxied to the inner side of each hemisphere, as shown in Figure 9b. Connecting wires are soldered at the edge of each plate and routed out of the tank through holes at the top and bottom. The top hole also serves as a port for filling the tank with liquid for testing. A data acquisition system from Tech4Imaging LLC, as shown in Figure 9c, is used to collect capacitance data.

The experiment is conducted with mineral oil instead of a real cryogenic liquid, such as oxygen, due to apparatus limitations at present. However, the relative permittivities of the two are 2.2 and 1.5, respectively, close enough for current purposes. The tank is filled with oil from the top in steps and capacitance data are recorded. The oil accumulates at the bottom of the tank, forming a stratified filling that is ‘gap’-centered. The 2-norm of the capacitance data is shown in Figure 9d, which exhibits a monotonic increase with mass fraction, confirming the proper functionality of the acquisition system. Finally, the capacitance data are fed to the machine learning model, trained with the same dataset as shown in Table 1, and mass fraction estimation is performed. The result is shown in Figure 9e in terms of percent estimation error, which exhibits a root mean squared error (RMSE) of 4.7 %, with a maximum of 10 % near the mid range. Although slightly degraded as compared to simulation results for the mild noise case shown in Figure 7, this result is highly promising. The extra error may have originated from the mismatches between measurement and simulation data, as observed by the differences between the capacitance norm curves in Figure 9d and Figure 6 for the stratified-gap cases, especially at low mass fractions. This difference may have originated from minor differences between the physical tank and the corresponding CAD model used for simulation, which can be mitigated by more careful design considerations.

In the future, more realistic experiments are needed considering the presence of various internal structures within the tank, such as diaphragms, pipe connections, and metallic guides. These are needed to be properly modeled in simulations during training data generation. Metallic guides can be left as floating or grounded, as discussed in [21]. However, it is suggested that they be grounded to prevent any undesired static electricity generation due to friction with the liquid. As such, this may cause reduced sensitivity for certain plate pairs, leading to performance degradation to some extent, which is subject to further analysis.

## 5. Discussion

This paper investigates a number of mass fraction estimation techniques based on capacitance data from a 12-plate spherical ECVT sensor for propellant mass gauging at micro-gravity conditions. Among the different techniques, the average-capacitance-based approach is the simplest; however, it possesses a peak error of 10% over the whole range, which is significant. The second technique, weighted average capacitance by channel type, can improve performance at lower fill levels but at the cost of poor performance at higher fill levels. The third technique, averaging the image generated by linear back projection algorithm, also performs poorly, peaking above 20% at certain ranges. However, this approach is often considered as a byproduct of the imaging step, which helps in visualizing the mass distribution in the RoI. The fourth technique, the capacitance-2-norm-based approach, shows similar performance to the average-capacitance-based approach. Lastly, the machine-learning-based approach shows by far the highest accuracy. With a good SNR, errors below 4% can be achieved across the whole measurement range and errors below 2% for fills of less than 50%. The experimental results confirm the applicability of this method subject to careful design considerations to minimize modeling errors.

Machine learning is a promising path forward for ECVT microgravity mass gauging, and further optimization may further increase the accuracy. It should be noted that the fill types analyzed here attempt to take into account possible instantaneous fluid states, including during sloshing, acceleration, or deceleration. Detailed fluid position information from mission planners may eliminate certain fill profiles, such as the core-annular configuration, and knowledge of spacecraft acceleration could select or eliminate stratified configurations, thereby tightening the training dataset. Fluid position data from imaging algorithms could also be combined with other approaches, as an input to the machine learning model, or as a first step in a multi-step averaging approach.

Further model optimization is warranted to increase mass fraction measurement accuracy. In addition, this sensor design should be tested in microgravity conditions to collect raw capacitance data on actual fluids, which can later be used in analysis and training.

## Figures and Tables

**Table 1:** Number of data points for different fill types.

Fill Type	Object Center Distance from Sensor Origin, *r*	Number of Directions/ Locations, (θ,ϕ), *L*	Mass Fraction Range	Number of Data Points, *N*	Total Number of Data Points, L×N
Stratified	N/A	26	0.0–1.0	100	2600
Core-annular	N/A	1	0.0–1.0	100	100
Core-annular, off-centered	1/8	32	0.0–0.62	60	1920
	1/4	32	0.0–0.36	40	1280
	3/8	32	0.0–0.21	20	640
	1/2	32	0.0–0.10	10	320
	5/8	32	0.0–0.04	5	160
Annular	N/A	1	0.0–1.0	100	100
Annular, off-centered	1/8	32	0.38–1.0	60	1920
	1/4	32	0.64–1.0	40	1280
	3/8	32	0.79–1.0	20	640
	1/2	32	0.90–1.0	10	320
Total	N/A	316	N/A	N/A	11,280

## Data Availability

Data used in this research is available upon request.
